# Evaluation of the Physicochemical and Biological Properties of Calcium-Silicate-Based Root-End Filling Materials

**DOI:** 10.3390/jfb17030131

**Published:** 2026-03-09

**Authors:** Asuka Aka, Takashi Matsuura, Atsutoshi Yoshimura

**Affiliations:** Department of Periodontology and Endodontology, Nagasaki University Graduate School of Biomedical Sciences, Nagasaki City 852-8588, Nagasaki, Japan; a-aka@nagasaki-u.ac.jp (A.A.); ayoshi@nagasaki-u.ac.jp (A.Y.)

**Keywords:** endodontic bioceramics, Bio-C repair, ProRoot MTA, human periodontal ligament cells, cytocompatibility, physicochemical properties, root-end filling materials

## Abstract

This study compared the physicochemical and biological properties of Bio-C Repair (BR), a new putty-type calcium silicate-based material, with ProRoot MTA (P) and Super-Bond (SB). Discs of the three materials were prepared. Human periodontal ligament cells were seeded onto the discs, and metabolic activity was assessed by MTT assay on days 7 and 28; cells without discs served as the negative control (NC). Moreover, the pH and calcium ion concentration of the eluate, the mass change, and the water sorption were investigated. On day 7, BR showed significantly lower cell activity than P and NC. However, by day 28, BR activity increased significantly, with no significant difference relative to other groups, whereas P activity was significantly suppressed relative to SB and NC. Physiochemically, BR maintained a significantly higher alkalinity (pH ~11.0) and greater calcium ion release than P throughout the 28 days. Furthermore, BR exhibited significant mass gain (15.7%) and the highest water sorption (15.4%), whereas P showed mass loss (−1.1%). Although the high pH of BR initially suppressed cell activity, it demonstrated favorable cytocompatibility by day 28. BR showed a significantly improved long-term cellular response compared to P, suggesting it is a promising alternative as a root-end filling material.

## 1. Introduction

The introduction of mineral trioxide aggregate (MTA) in the early 1990s marked a significant paradigm shift in endodontic therapy [1]. ProRoot MTA (P) was the first calcium silicate-based cement approved for clinical use, originally designed to overcome the limitations of traditional materials like amalgam, IRM, and Super-EBA [2]. Over the past three decades, it has become widely accepted as the “gold standard” for retrograde filling in endodontic microsurgery, primarily due to its excellent biocompatibility and its unique ability to induce hard tissue formation [3,4]. However, it has drawbacks, including poor handling properties and susceptibility to washout [5,6]. To overcome these limitations, various products have been developed in recent years [7,8].

To address these long-standing clinical challenges, Bio-C Repair (BR; Angelus, Londrina, PR, Brazil) was recently introduced as a new generation of bioceramic materials [9]. Unlike traditional MTA, which requires manual mixing of powder and liquid, BR is a premixed, putty-type calcium silicate-based cement that can be applied immediately [9]. This “ready-to-use” formulation significantly enhances clinical efficiency by providing consistent viscosity and eliminating the potential for mixing errors [10]. Chemically, it utilizes zirconium oxide as a radiopacifier instead of bismuth oxide, a modification specifically intended to prevent the crown discoloration often associated with conventional MTA [11]. Furthermore, its non-aqueous organic medium allows the material to remain stable in the syringe while ensuring a controlled setting reaction upon contact with moisture in the periradicular tissues [12]. While these advancements offer promising clinical benefits, scientific data regarding their long-term biological interaction with human periodontal tissues are still accumulating.

Meanwhile, alongside traditional cements, Super-Bond (SB; Sun Medical, Moriyama, Japan) (marketed as C&B-Metabond in the USA), a 4-META/MMA-TBB-based adhesive resin, has been clinically utilized in surgical endodontics in Japan since the pre-MTA era [13]. Known for its high bond strength to both dentin and various dental materials, SB provides an effective hermetic seal by forming a hybrid layer [14]. The tri-n-butylboron catalyst in SB can polymerize even in the presence of moisture [15], and its markedly lower radical production rate allows greater cell growth than with the PMMA/MMA-BPO resin and enhanced osteoblastic differentiation [16,17,18,19]. These unique properties made it a preferred choice for retrograde filling and root repair.

Given that many of these contemporary materials have emerged within the last decade, comprehensive evaluations of their properties remain limited. Furthermore, the biological properties of these endodontic bioceramics are strongly influenced by components released during and after setting, as well as by the resulting pH changes in the surrounding environment. In general, calcium silicate-based materials generate calcium hydroxide during hydration, which is highly alkaline. While this alkalinity and the release of calcium ions contribute to antibacterial activity and hard-tissue induction [20], excessively high pH, material dissolution (mass change), or water sorption may cause cellular irritation or affect the material’s sealing ability [21,22].

Consequently, the primary objective of this study was to investigate the cytocompatibility of BR, P, and SB using human periodontal ligament-derived cells (hPDLCs). The secondary objectives were to evaluate: (1) the pH and calcium ion concentration of the eluate, and (2) the mass change and water sorption properties of these materials. The null hypotheses tested were that no significant differences would be observed among BR, P, SB, and the negative control group regarding their cytocompatibility, pH and calcium ion release, or mass change and water sorption

## 2. Materials and Methods

**Figure 1:**
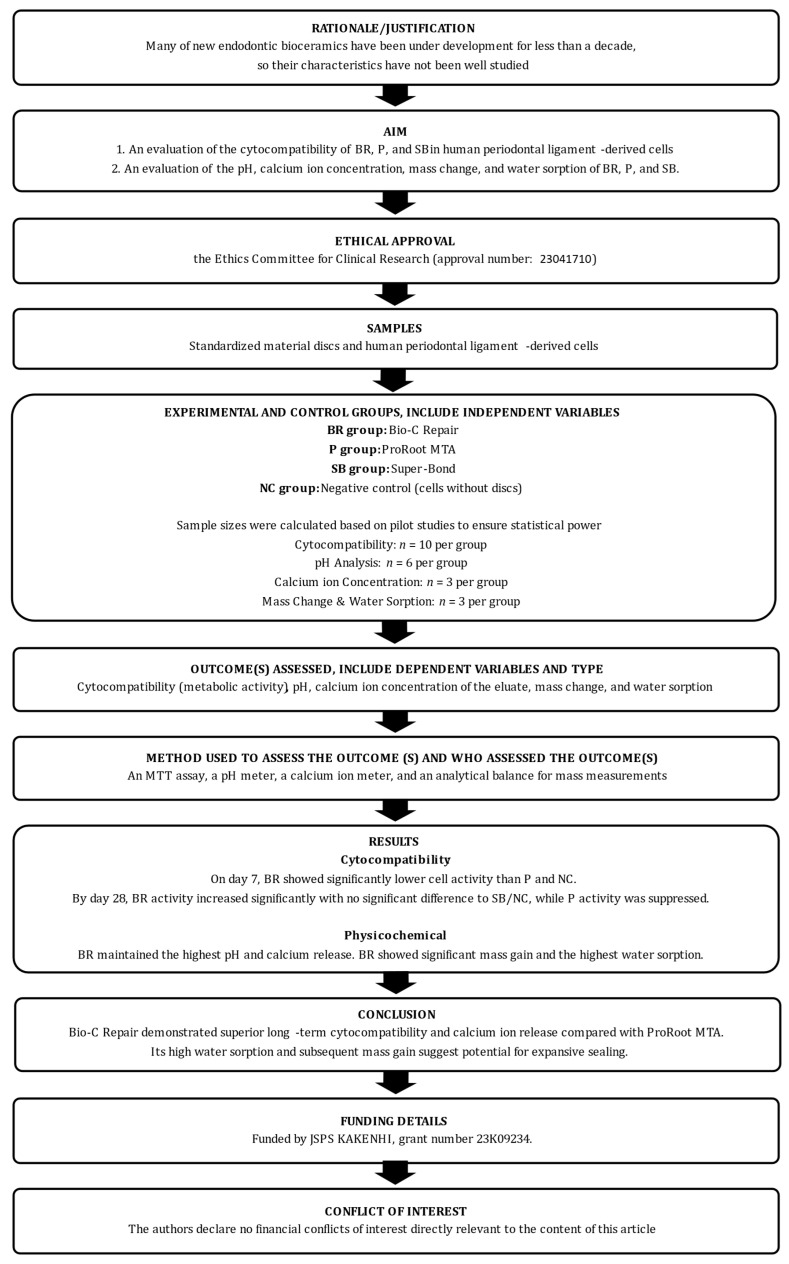
The PRILE 2021 flowchart of this study.

Reporting of this laboratory study was conducted in accordance with the Preferred Reporting Items for Laboratory studies in Endodontology (PRILE) 2021 guidelines [23,24]. The procedural steps are illustrated in the PRILE 2021 flowchart (Figure 1), and the comprehensive PRILE 2021 checklist is provided in Supplementary Table S1.

### 2.1. hPDLCs

Teeth were obtained from patients who presented to our hospital for tooth extraction and provided verbal consent for this study. Healthy human premolars or third molars with no evidence of periodontitis, pericoronitis, or periapical pathology were included, with one tooth used for each independent cell isolation. The preparation of hPDLCs was performed as previously described [25].

### 2.2. Preparation of Bioceramic Discs

BR, P, and SB were prepared according to the manufacturers’ instructions, packed into molds (inner diameter = 8 mm, thickness = 1 mm), and incubated in a 5% CO_2_ incubator at 37 °C and 100% humidity for 48 h to form discs (*n* = 10; Table 1). This duration was determined based on preliminary experiments that showed 24 h of incubation resulted in material fragility and a risk of deformation during handling.

### 2.3. Cytocompatibility

For the biological evaluation, each material disc was placed at the bottom of a 48-well plate, and hPDLCs were seeded at 100,000 cells/well. The random order was generated by A.A. in accordance with previously reported protocols [25]. The cells were maintained in 500 μL of Dulbecco’s Modified Eagle Medium supplemented with 10% Fetal bovine serum and 1% Penicillin-streptomycin. To determine the metabolic activity, a blinded investigator conducted the MTT (3-[4,5-dimethylthiazol-2-yl]-2,5-diphenyl tetrazolium bromide) assay on days 7 and 28, following previously established protocols [25]—cells grown in the absence of any disc served as the negative control (NC). The relative cell viability was calculated as a percentage of the NC group using the following formula:

$$\text{Relative}\;\text{cell}\;\text{viability}\;\left(\%\right)=\frac{{OD}_{\text{sample}}}{{\text{Mean}\;OD}_{NC}}\;\times \;100$$

where *OD_sample_* is the absorbance of the experimental group, and *Mean OD_NC_* is the mean absorbance of the NC group at day 7.

### 2.4. pH and Calcium Ion Analysis of the Eluate

To evaluate pH and calcium ion release in the surrounding environment, bioceramic discs (diameter = 8 mm, thickness = 1 mm) were prepared as described in Section 2.2. After setting for 48 h, each disc was immersed in 500 μL of deionized water in a 48-well plate. They were stored in a 5% CO_2_ incubator at 37 °C and 100% humidity for 28 days. The water was replaced every 2 to 3 days until day 28, and the eluate pH and calcium ion were measured at day 3, 5, 7, 10, 12, 14, 17, 19, 21, 24, 26, and 28 with a pH meter (S2K333, Toyorika, Tokyo, Japan) (*n* = 6) and a calcium ion meter (LAQUAtwin Ca-11; Horiba, Kyoto, Japan) (*n* = 3)—deionized water without a disc served as NC.

### 2.5. Mass Change and Water Sorption

Initial Mass (*M*_0_): The discs were weighed immediately after the 48-h setting period using an analytical balance (Sartorius ED124S, Germany). Each disc was then immersed in 500 μL of deionized water in a 48-well plate and stored in a 5% CO_2_ incubator at 37 °C and 100% humidity for 28 days. The water was replaced every 2 to 3 days until day 28.

Final and Desiccated Mass (*M_f_* and *M_d_*): At day 28, the discs were removed from the liquid, gently blotted with filter paper to remove excess surface moisture, and weighed to determine the final wet mass (*M_f_*) (*n* = 3). Subsequently, the discs were replaced in a 5% CO_2_ incubator at 37 °C and 100% humidity for 48 h, after which the final dry mass (Md) was recorded.

Calculations: The percentage of mass change and water sorption were calculated using the following formulas:

$$\text{Mass}\;\text{Change}\;\left(\%\right)=\frac{M_{d}-M_{0}}{M_{0}}\;\;\times 100$$

$$\text{Water}\;\text{Sorption}\;\left(\%\right)=\frac{M_{f}-M_{d}}{M_{d}}\;\times 100$$

### 2.6. Statistical Analysis

Because equal variances were not assumed among some experimental groups, pairwise comparisons were performed using Welch’s *t*-test with Bonferroni correction to control the family-wise error rate. Statistical evaluations among the four experimental groups (BR, P, SB, and NC) or three experimental groups (BR, P, and SB) were conducted using Welch’s *t*-test. To maintain the family-wise error rate, *p*-values were corrected for multiple comparisons using the Bonferroni method, with the threshold for statistical significance set at α = 0.008 (0.05/6) or α = 0.0167 (0.05/3). Additionally, a paired *t*-test was used to compare the relative metabolic activity between day 7 and day 28 within each group, with *α* = 0.05. Differences in pH and calcium ion release were analyzed using two-way repeated measures ANOVA. Post hoc multiple comparisons between materials were conducted using the Tukey–Kramer test with a significance level of α = 0.05. This method was chosen to control the family-wise error rate across all pairwise comparisons. All computational analyses were performed using IBM SPSS Statistics software, version 27.0 (IBM Corp., Armonk, NY, USA).

### 2.7. Sample Size

Pilot studies were conducted to calculate the sample size for each experiment. The data from the pilot studies are presented in Figures S1–S4 and in Tables S2–S5. The sample sizes were calculated based on the results of the preliminary experiments. The sample size of the experiment to evaluate the cytocompatibility of BR, P, and SB was calculated to detect a difference between the BR group (mean and standard deviation [SD]: 0.67 [0.52]) and the NC group (mean [SD]: 1.50 [0.05]) with a power of 80% at a 2-tailed significance level of 0.008. Ten samples were used, with a 20% dropout rate. The sample size of the experiment to evaluate the pH of the eluate of BR, P, and SB was calculated to detect a difference between the BR group at day 3 (mean and standard deviation [SD]: 11.4 [0.2]) and the NC group at day 3 (mean [SD]: 8.7 [0.1]) with a power of 90% at a 2-tailed significance level of 0.008. Six samples were used, with a 20% dropout rate. The sample size of the experiment to evaluate the calcium ion concentration of the eluate of BR, P, and SB was calculated to detect a difference between the BR group (mean and standard deviation [SD]: 81.5 [0.71]) and the NC group (mean [SD]: 0 [0]) with a power of 90% at a 2-tailed significance level of 0.008. Three samples were used, with a 20% dropout rate. The sample size of the experiment to evaluate the mass change of BR, P, and SB was calculated to detect a difference between the BR group (mean [SD]: 14.4% [0.3]) and the SB group (mean [SD]: 1.8% [0.2]) with a power of 90% at a 2-tailed significance level of 0.0167. Three samples were used, with a 20% dropout rate.

## 3. Results

### 3.1. Cytocompatibility

**Figure 2:**
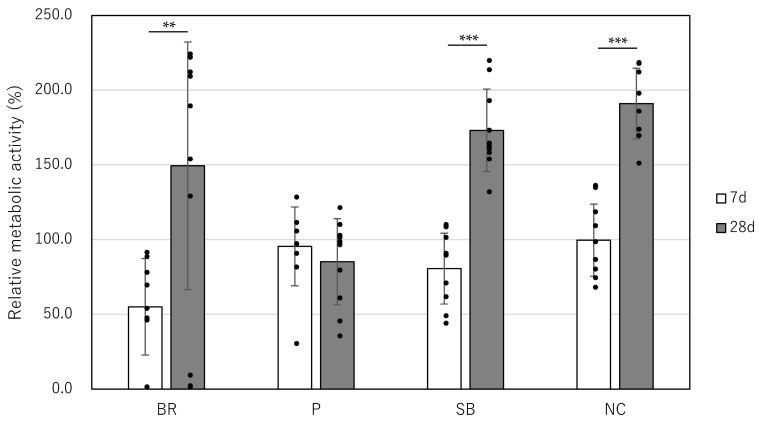
Cytocompatibility of root-end filling materials in hPDLCs. After 7 days and 28 days of incubation with bioceramic materials. **: *p* < 0.01; ***: *p* < 0.001; BR: Bio-C Repair; P: ProRoot MTA; SB: Super-Bond; NC: negative control.

On day 7, the relative metabolic activity [mean (SD)] was 55.0 (32.3) for the BR group, 95.5 (26.4) for the P group, 80.6 (23.7) for the SB group, and 99.6 (24.2) for the NC group (Table 2 and Figure 2). Detailed statistical distribution, including individual data points and standard deviations, has been provided to ensure full transparency of the observed variations. Significant differences were observed between the BR and P groups (*p* = 0.005) and between the BR and NC groups (*p* = 0.002). On day 28, the absorbance was 149.4 (82.8) for the BR group, 85.2 (28.8) for the P group, 173.1 (27.6) for the SB group, and 191.0 (23.6) for the NC group (Table 3 and Figure 2). Significant differences were found between the P and SB groups, as well as between the P and NC groups (*p* < 0.001).

### 3.2. The pH and Calcium Ion Concentration of the Eluate

**Figure 3:**
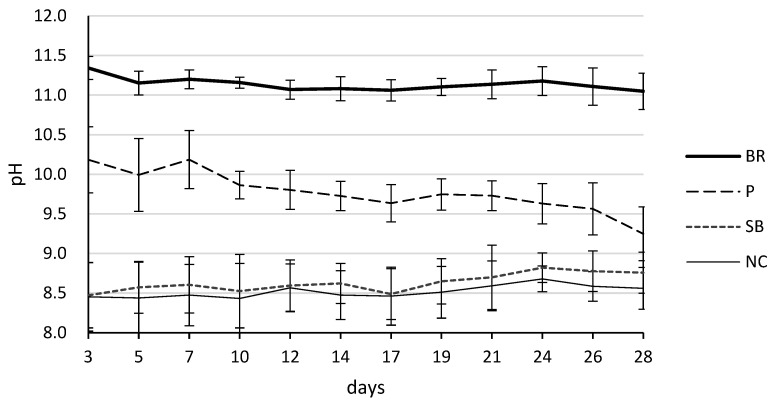
Changes in pH over time across different treatment groups. The pH values were measured on days 3, 5, 7, 10, 12, 14, 17, 19, 21, 24, 26, and 28. Groups include BR (solid line), P (long dashed line), SB (dotted line), and NC (thin solid line). Data are presented as mean ± standard deviation (SD).

The pH levels of the eluates for each material over the 28-day period are summarized in Table 4 and Figure 3. Two-way repeated-measures ANOVA revealed a significant interaction between material type and time (*p* < 0.001). BR maintained the highest alkalinity among all groups, with pH values remaining stable between approximately 11.0 and 11.3 throughout the experimental period. Post hoc multiple comparisons using the Tukey–Kramer test confirmed that the pH of the BR group was significantly higher than that of the P, SB, and NC groups at all time points (*p* < 0.001). In contrast, the P group initially had a pH of approximately 10.2 on day 3, which decreased to 9.2 by day 28 (*p* < 0.001). Although the pH of the P group remained significantly higher than that of the SB and NC groups (*p* < 0.001), it demonstrated a distinct downward trend over time. The SB and NC groups exhibited stable and near-neutral pH levels, ranging from 8.4 to 8.8, with no significant differences observed between these two groups throughout the 28 days.

**Figure 4:**
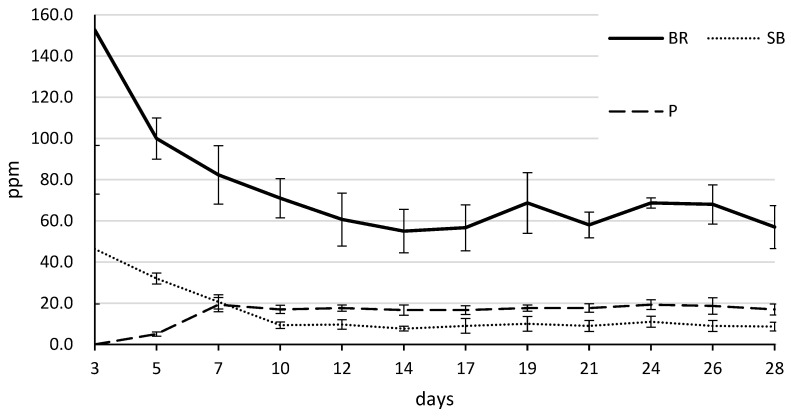
Changes in calcium ion over time across different treatment groups. Calcium ion release was measured on days 3, 5, 7, 10, 12, 14, 17, 19, 21, 24, 26, and 28. Groups include BR (solid line), P (long dashed line) and SB (dotted line). Data are presented as mean ± standard deviation (SD).

The calcium ion concentrations released from the tested materials over 28 days are presented in Table 5 and Figure 4. A two-way repeated-measures ANOVA showed a significant interaction between material type and time (*p* < 0.001). BR exhibited the highest calcium ion release throughout the experimental period, characterized by an initial peak of 152.7 (56.0) ppm at day 3, followed by a gradual decrease to 57.0 (10.4) ppm by day 28. Post hoc multiple comparisons using the Tukey–Kramer test revealed that the cumulative calcium release of the BR group was significantly higher than that of the P, SB, and NC groups (*p* < 0.001). P showed a different release pattern; no calcium ions were detected at day 3, but the concentration increased to 19.3 (3.5) ppm at day 7 and remained stable until day 28. The SB group showed a moderate initial release of 46.3 (26.6) ppm at day 3, which subsequently declined to 8.7 (2.1) ppm by day 28.

### 3.3. Mass Change and Water Sorption

**Figure 5:**
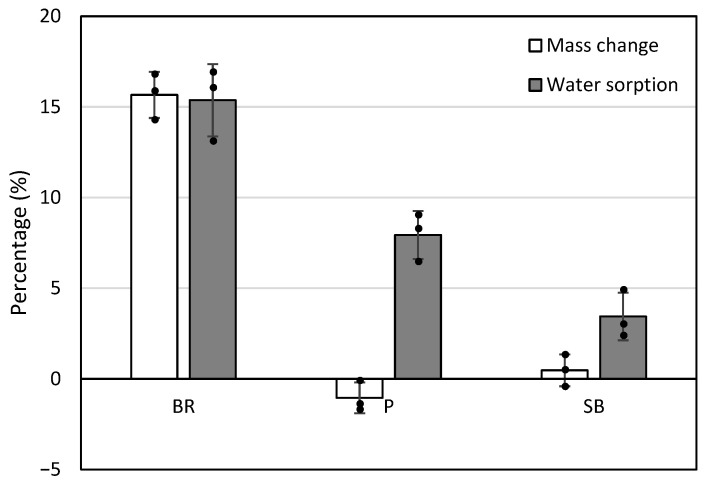
Mass change and water sorption of the tested materials. The bars represent the mean percentage change in mass (light gray) and water sorption (dark gray) for BR, P, and SB after 28 days of immersion in deionized water. Error bars indicate the standard deviation, and black circles represent individual data points (*n* = 3). Positive values in mass change indicate a weight gain, while negative values indicate material dissolution (mass loss).

The results for mass change and water sorption after 28 days of immersion are summarized in Table 6 and Figure 5. Mass change: BR exhibited a significant mass gain of 15.7% (SD: 1.3), which was statistically higher than both P at −1.1% (SD: 0.8) and SB at 0.5% (SD: 0.9) (*p* < 0.008). While BR and SB showed positive values (weight gain), P was the only material to demonstrate a negative value, indicating material dissolution or mass loss. Water sorption: BR showed the highest water sorption at 15.4% (SD: 2.0). In contrast, the water sorption for P and SB was 7.9% (SD: 1.3) and 3.4% (SD: 1.3), respectively. BR showed significantly higher water sorption compared to P and SB.

Regarding the long-term cellular response, it should be noted that the metabolic activity observed on day 28 was interpreted with caution. While the P group showed a significant decrease in relative activity compared to the NC and SB groups, this result may be influenced not only by the material’s potential toxicity but also by biological factors such as contact inhibition or density-dependent metabolic shifts occurring in prolonged cultures. Therefore, the day 28 data were used as a comparative indicator of metabolic trends rather than an absolute measure of cell viability.

## 4. Discussion

The clinical success of root-end filling materials depends on their biological interaction with periradicular tissues and their physical stability. In this study, we evaluated the physicochemical and biological properties of Bio-C Repair, a premixed putty-type bioceramic, compared with the gold standard ProRoot MTA and the traditional adhesive resin Super-Bond. The null hypotheses were rejected, as significant differences were observed across all tested parameters.

### 4.1. Biological Response and the Role of pH

The biological response of hPDLCs appears to be closely linked to the material’s chemical dynamics. In the BR group, the inclusion of PEG may have promoted rapid water infiltration, thereby accelerating the hydration of calcium silicates. This physicochemical process is thought to contribute to the sustained high alkalinity (pH ~11.0) and the substantial initial release of calcium ions (152.7 ppm at day 3) observed in our study. While these properties are beneficial for bioactivity, the extreme pH levels may have initially caused cellular stress, potentially explaining the significant suppression of metabolic activity on day 7. However, as the material stabilized, this high bioactivity likely contributed to the significant recovery of cell activity by day 28.

This recovery suggests that once the initial high-intensity reaction stabilizes, the material provides a favorable environment for sustained cellular activity. These results are consistent with previous findings that BR exhibits favorable cytocompatibility while maintaining a high alkaline environment and substantial calcium ion release, both of which are attributable to its hydrated calcium silicate composition.

A key finding was the temporal change in cytocompatibility for the BR group. On day 7, BR showed significantly lower cell activity than P and NC. This initial suppression may be associated with the physicochemical changes observed in the material’s interaction with the hydrophilic vehicle, polyethylene glycol (PEG), present in BR. As a hydrophilic agent, PEG promotes rapid moisture infiltration into the material, thereby accelerating the hydration of calcium silicates [26]. Consequently, BR maintained a high alkalinity, with a pH exceeding 11.0 from the early stages (Table 4 and Figure 3), which is thought to temporarily inhibit metabolic activity and cell proliferation in hPDLCs. Furthermore, the accelerated hydration facilitated by PEG resulted in a rapid and substantial release of calcium ions during the initial period, reaching 152.7 ppm at day 3 (Table 5 and Figure 4). Such excessive fluctuations in ion concentration and extreme pH levels may have acted as sources of initial cellular stress. While an alkaline environment is beneficial for its antibacterial effects and for inducing hard tissue formation, these results suggest that BR’s high-intensity chemical activity initially outweighs its cytocompatibility. By day 28, however, the metabolic activity of the BR group increased significantly, with no significant difference relative to SB or NC. This recovery suggests that once the initial high-intensity reaction stabilizes, the material provides a favorable environment for sustained cellular activity. These results are consistent with previous findings that BR exhibits favorable cytocompatibility while maintaining a high alkaline environment and substantial calcium ion release, attributable to its hydrated calcium silicate composition [27,28,29]. The sustained high pH observed in our study further confirms its potent bioactive potential. The sustained high pH observed in our study further suggests its potent bioactive potential. Although this study did not perform a direct interfacial analysis, recent research using energy-dispersive X-ray spectroscopy (EDX) has demonstrated that bioceramic materials facilitate the migration of bioactive elements, such as calcium, phosphorus, and silicon, toward the dentin-sealer interface. This process promotes the formation of hydroxyapatite and enhances the bioactivity of the dentin interface. Such experimental evidence from the literature supports the proposed mechanism that the high alkalinity and substantial calcium ion release observed in BR contribute to effective interfacial remineralization and biological integration [30].

Regarding the day 28 data, although high variance in metabolic activity was observed in the BR group, statistical validity was maintained by using Welch’s *t*-test, which does not assume equal variances between groups. This high standard deviation (2.11 ± 1.17) likely reflects varying individual cell recovery rates following the initial alkaline stress and potential inconsistencies in the material’s surface microtopography. Materials with high water sorption, such as BR (15.4%), are prone to microscopic surface collapse or non-uniform expansion during immersion, resulting in variations in surface area and microstructure in contact with cells [31,32,33]. Furthermore, as the hydrophilic vehicle PEG dissolves, it can create irregular porous structures within the material, leading to local pH fluctuations and calcium ion release, which further contribute to the observed variability in cell activity [34,35,36,37].

In contrast to BR, the metabolic activity in the P group did not increase significantly between days 7 and 28. This result is consistent with the previous study by Camilleri et al. [38]. It has been suggested that bismuth oxide, used as a radiopacifier in P, may possess potential cytotoxicity [39,40,41]. Its exposure or elusion during long-term immersion might have continuously suppressed the cells’ metabolic activity.

### 4.2. Physicochemical Properties: BR vs. P

The physical dynamics of BR showed different trends compared to P. BR exhibited a 15.7% increase in mass and 15.4% in water sorption after 28 days. These values are notably higher than those of traditional MTA, primarily due to the inclusion of hydrophilic excipients, such as PEG, in the premixed formulation [26,42]. Furthermore, the physical stability of the materials, represented by mass change and water sorption, may be linked to their clinical performance. The significant mass gain in BR (15.7%) due to high water sorption (15.4%) suggests a volumetric expansion that could enhance the hermetic seal against dentinal walls.

While high solubility is typically viewed negatively, the “mass gain” observed here indicates that water sorption and subsequent volumetric expansion outweighed the dissolution of material components. This physical behavior, combined with the continuous elution of ions enabled by the constant concentration gradient in the periradicular environment, is thought to help ensure that BR maintains its bioactive potential over the long term. However, this physical behavior must be interpreted with caution. High water sorption can potentially lead to increased porosity and a decrease in the material’s cohesive strength over time. The dissolution of hydrophilic components like PEG may create microscopic voids, which, while facilitating ion release, could also compromise the long-term mechanical stability and dimensional integrity of the material under clinical loading. Therefore, although the expansive properties are promising for sealing, they represent a trade-off that requires further investigation regarding long-term structural durability.

In addition, the experimental protocol of replacing the deionized water every 2 to 3 days may have further influenced these results. This procedure was designed to simulate the dynamic clinical environment in which periradicular tissues are continuously replenished with interstitial fluid [43,44]. By preventing chemical saturation of the medium, a constant concentration gradient was maintained, likely encouraging the continuous elution of ions and the sustained high alkalinity observed in the BR group. By preventing chemical saturation of the medium, a constant concentration gradient was maintained, likely encouraging the continuous elution of ions and the sustained high alkalinity observed in the BR group. However, it is important to note that using deionised water as the immersion medium may artificially amplify these values compared to those in a clinical environment. Unlike deionised water, biological fluids possess inherent buffering capacities that would likely moderate extreme pH shifts and influence ion solubility. Therefore, while these results effectively demonstrate the inherent bioactive potential and ion-releasing capacity of BR, direct extrapolation to clinical concentrations should be made with caution, as the periradicular tissues may attenuate these chemical changes. While this environmental refreshment provides a more realistic representation of long-term bioactivity, the resulting high-intensity chemical environment may have contributed to the cellular stress reflected in the day 7 MTT results.

### 4.3. The Significance of SB

Including SB provides a contrast, representing a “sealing by adhesion” philosophy rather than “bioactive mineralization”. SB demonstrated excellent, stable cytocompatibility from day 7 to day 28, consistent with its long-standing clinical success since the pre-MTA era. Its lack of pH change and calcium release confirms its role as a chemically stable, non-interactive barrier. Although a minor initial release of calcium ions (46.3 ppm) was detected in the SB group at day 3 (Table 5), this is likely attributable to trace impurities remaining in the raw materials—such as polymers or radiopaque fillers—rather than intentional bioactive additives. This interpretation is supported by the rapid decrease in calcium concentration over time and by information from the manufacturer regarding similar trace detections in related resin products. In clinical scenarios such as intentional replantation—frequently practiced in Japan—the immediate, high-strength hermetic seal provided by SB via the formation of a hybrid layer remains highly effective [45]. While SB lacks the bioactive properties of calcium silicates, its ability to polymerize in moist environments using the TBB catalyst ensures reliable performance in surgical endodontics.

### 4.4. Study Limitations

This study has limitations. First, using a disc-shaped model may not fully replicate the complex 3D geometry and limited moisture availability of an actual root canal. Additionally, using deionized water as the immersion medium, rather than a simulated body fluid, may have artificially elevated pH and ion release values due to its lack of buffering capacity found in clinical environments. While the high water sorption of BR may contribute to an improved apical seal through hygroscopic expansion, its effect of significant water uptake on long-term dimensional stability and mechanical strength remains unclear. Further long-term studies are required to evaluate these effects.

Moreover, this study evaluated cytocompatibility primarily through metabolic activity using the MTT assay. While metabolic activity is a crucial indicator of early cellular response, it may not directly reflect long-term cell viability or proliferation. Regarding the long-term cellular response, we acknowledge that the MTT assay results at 7 and 28 days carry inherent uncertainties. At later time points, such as day 28, contact inhibition and density-dependent metabolic alterations may confound the interpretation of MTT results. Specifically, the slight decrease in relative metabolic activity observed in the P group at day 28 compared to day 7 highlights a potential limitation of the MTT assay in long-term cultures. At later time points, factors such as contact inhibition or reduced formazan diffusion through multilayered cell sheets may influence the readings, potentially leading to an underestimation of true cell viability. The lack of complementary assays, such as DNA quantification or Live/Dead staining at these later stages, is a limitation of this study.

Furthermore, the present study did not directly evaluate the contact angle or surface wettability of the materials. These parameters are closely related to cell attachment and biological behavior on the material surface. While the high water sorption (15.4%) observed in BR in this study reflects its hydrophilic composition, such as PEG, future research incorporating precise analysis of surface energy will provide more detailed insights into the interfacial interactions between these materials and cells.

Given that calcium silicate-based materials are designed to be bioactive, the lack of assessment for mineralized tissue markers—such as alkaline phosphatase (ALP) activity or the expression of osteogenic genes (e.g., RUNX2, OCN)—is another significant limitation. Future research should incorporate these markers and multiple viability/cytotoxicity assays to comprehensively evaluate long-term cellular responses, differentiation, and the potential of Bio-C Repair to induce hard tissue formation in a clinical context. Furthermore, the sample size for the evaluation of calcium ion release, mass change, and water sorption was limited to *n* = 3 per group. Although these sample sizes were determined based on preliminary power calculations intended to detect significant differences, the small number of samples may limit the representativeness and generalizability of the results. Future studies with a larger sample size are necessary to confirm the robustness of these physicochemical findings and to provide a more comprehensive characterization of the materials’ long-term behavior. Furthermore, the high variability in metabolic activity observed in the BR group at day 28 should be acknowledged. This variability likely reflects inconsistencies in the material’s surface characteristics and localized chemical changes resulting from the dissolution of the hydrophilic vehicle over time. Lastly, in vivo studies are necessary to evaluate the material’s ability to induce actual hard-tissue formation.

## 5. Conclusions

Bio-C Repair demonstrated favorable long-term cytocompatibility and calcium ion release compared with ProRoot MTA, despite an initial suppressive effect due to its high alkalinity. Its high water sorption and subsequent mass gain suggest the potential for expansive sealing. These findings indicate that BR is a promising, user-friendly alternative to traditional MTA for surgical endodontic procedures.

## Figures and Tables

**Table 1:** Materials used in this experiment.

Materials (Abbr.)	Manufactures	Composition
Bio-C Repair (BR)	Angelus (Brazil)	Paste: calcium silicates, silicon dioxide, zirconium oxide, polyethylene glycol, etc.
ProRoot MTA (P)	Dentsply Sirona (Charlotte, NC, USA)	Powder: calcium oxide, bismuth oxide, silicon dioxide, aluminum oxide, etc. Liquid: purified water, plasticizer
Super-Bond (SB)	Sun Medical (Japan)	Monomer: MMA, 4-META, etc. Powder: PMMA, etc. Catalyst: Tri-n-butylboron partial oxide, etc.

**Table 2:** The relative metabolic activity determined by the MTT assay after 7 days of incubation with root-end filling materials.

	1	2	3	4	5	6	7	8	9	10	Mean (SD)
BR	1.4	78.0	53.9	69.5	47.5	72.3	1.4	88.7	91.5	46.1	55.0 (32.3) ^a^
P	105.7	97.2	97.2	90.8	128.4	114.9	30.5	97.2	111.3	81.6	95.5 (26.4) ^b^
SB	109.9	108.5	70.9	44.0	101.4	80.9	90.8	61.7	89.4	48.9	80.6 (23.7) ^ab^
NC	118.4	98.6	86.5	74.5	134.8	90.1	136.2	80.1	109.2	68.1	99.6 (24.2) ^b^

BR: Bio-C Repair; P: ProRoot MTA; SB: Super-Bond; NC: Negative Control. Different lowercase letters indicate significant differences (*p* < 0.008).

**Table 3:** The relative metabolic activity determined by the MTT assay after 28 days of incubation with root-end filling materials.

	1	2	3	4	5	6	7	8	9	10	Mean (SD)
BR	2.1	143.3	209.2	129.1	224.1	212.1	9.2	222.0	189.4	153.9	149.4 (82.8) ^ab^
P	98.6	101.4	61.0	96.5	102.8	79.4	109.9	45.4	35.5	121.3	85.2 (28.8) ^a^
SB	153.9	219.9	173.0	131.9	192.9	164.5	213.5	161.0	162.4	158.2	173.1 (27.6) ^b^
NC	173.8	209.9	218.4	151.1	197.9	212.1	217.7	185.8	173.8	169.5	191.0 (23.6) ^b^

BR: Bio-C Repair; P: ProRoot MTA; SB: Super-Bond; NC: Negative Control. Different lowercase letters indicate significant differences (*p* < 0.008).

**Table 4:** pH in different samples over a 28-day storage period.

Day	3	5	7	10	12	14	17	19	21	24	26	28
BR	11.3 (0.1)	11.2 (0.2)	11.2 (0.1)	11.2 (0.1)	11.1 (0.1)	11.1 (0.2)	11.1 (0.1)	11.1 (0.1)	11.1 (0.2)	11.2 (0.2)	11.1 (0.2)	11.0 (0.2)
P	10.2 (0.4)	10.0 (0.5)	10.2 (0.4)	9.9 (0.2)	9.8 (0.2)	9.7 (0.2)	9.6 (0.2)	9.7 (0.2)	9.7 (0.2)	9.6 (0.3)	9.6 (0.3)	9.2 (0.3)
SB	8.5 (0.4)	8.6 (0.3)	8.6 (0.4)	8.5 (0.5)	8.6 (0.3)	8.6 (0.3)	8.5 (0.3)	8.6 (0.3)	8.7 (0.4)	8.8 (0.2)	8.8 (0.3)	8.8 (0.3)
NC	8.5 (0.4)	8.4 (0.5)	8.5 (0.4)	8.4 (0.4)	8.6 (0.3)	8.5 (0.3)	8.5 (0.4)	8.5 (0.3)	8.6 (0.3)	8.7 (0.2)	8.6 (0.2)	8.6 (0.3)

BR: Bio-C Repair; P: ProRoot MTA; SB: Super-Bond; NC: negative control.

**Table 5:** Concentrations of calcium ion (ppm) in different samples over a 28-day storage period.

Day	3	5	7	10	12	14	17	19	21	24	26	28
BR	152.7 (56.0)	100.0 (10.0)	82.3 (14.2)	71.0 (9.5)	60.7 (12.9)	55.0 (10.5)	56.7 (11.2)	68.7 (14.7)	58.0 (6.2)	68.7 (2.5)	68.0 (9.5)	57.0 (10.4)
P	0.0 (0.0)	5.0 (1.0)	19.3 (3.5)	17.0 (2.0)	17.7 (1.5)	16.7 (2.5)	16.7 (2.1)	17.7 (1.5)	17.7 (2.1)	19.3 (2.3)	18.7 (4.0)	17.0 (2.6)
SB	46.3 (26.6)	32.0 (2.6)	20.7 (3.5)	9.3 (1.5)	9.7 (2.3)	7.7 (1.2)	9.0 (3.6)	10.0 (3.6)	9.0 (2.6)	11.0 (2.6)	9.0 (2.6)	8.7 (2.1)
NC	0.0 (0.0)	0.0 (0.0)	0.0 (0.0)	0.0 (0.0)	0.0 (0.0)	0.0 (0.0)	0.0 (0.0)	0.0 (0.0)	0.0 (0.0)	0.0 (0.0)	0.0 (0.0)	0.0 (0.0)

BR: Bio-C Repair; P: ProRoot MTA; SB: Super-Bond; NC: negative control.

**Table 6:** Mass change (%) and water sorption (%) of the tested materials after 28 days of immersion.

Material	Property	1	2	3	Mean (SD)
BR	Mass change (%)	16.8	15.9	14.3	15.7 (1.3) ^a^
	Water sorption (%)	16.9	13.1	16.1	15.4 (2.0) ^a^
P	Mass change (%)	−1.7	−0.1	−1.4	−1.1 (0.8) ^b^
	Water sorption (%)	9.0	8.3	6.5	7.9 (1.3) ^ab^
SB	Mass change (%)	1.3	−0.4	0.5	0.5 (0.9) ^b^
	Water sorption (%)	4.9	2.4	3.0	3.4 (1.3) ^b^

BR: Bio-C Repair; P: ProRoot MTA; SB: Super-Bond. Different lowercase letters indicate significant differences (*p* < 0.0167).

## Data Availability

The data presented in this study are available on request from the corresponding author.

## Supplementary Materials

The following supporting information can be downloaded at: https://www.mdpi.com/article/10.3390/jfb17030131/s1, Figure S1: Cytocompatibility of endodontic bioceramics in hPDLCs; Figure S2: Changes in pH over time across different treatment groups; Figure S3: Changes in calcium ion over time across different treatment groups; Figure S4: Mass change and water sorption of the tested materials; Table S1: PRILE 2021 checklist; Table S2: Absorbance determined by the MTT assay after 7 days of incubation with root-end filling materials; Table S3: pH in different samples over a 28-day storage period; Table S4: Concentrations of calcium ion (ppm) in different samples over a 28-day storage period; Table S5: Mass change (%) and Water sorption (%) of the tested materials after 28 days of immersion.

## Abbreviations

The following abbreviations are used in this manuscript:

BR	Bio-C Repair
hPDLCs	Human periodontal ligament-derived cells
MTA	Mineral trioxide aggregate
MTT	3-[4,5-dimethylthiazol-2-yl]-2,5 diphenyl tetrazolium bromide
NC	Negative control
P	ProRoot MTA
PRILE	Preferred Reporting Items for Laboratory Studies in Endodontology
SB	Super-Bond
SD	Standard deviation
