# Supplementary material for: Evaluation of the Physicochemical and Biological Properties of Calcium-Silicate-Based Root-End Filling Materials
Source: J Funct Biomater. 2026 Mar 9;17(3):131. doi: 10.3390/jfb17030131 (PMC13028352; doi:10.3390/jfb17030131)
Supplement: Supplementary file 1 [file jfb-17-00131-s001.zip › Figure S1-4.pdf]

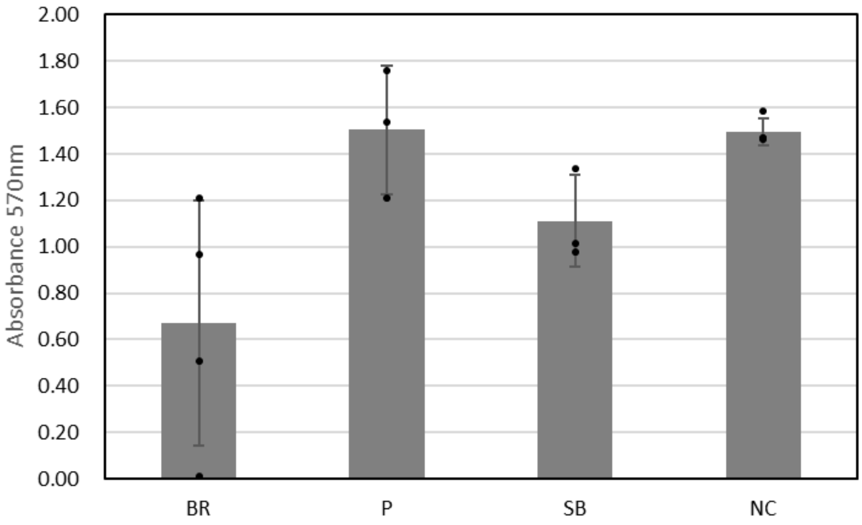

Figure S1. Cytocompatibility of endodontic bioceramics in hPDLCs. After 7 days of incubation with bioceramic materials. BR: Bio-C Repair; P: ProRoot MTA; SB: Super-Bond; NC: negative control.

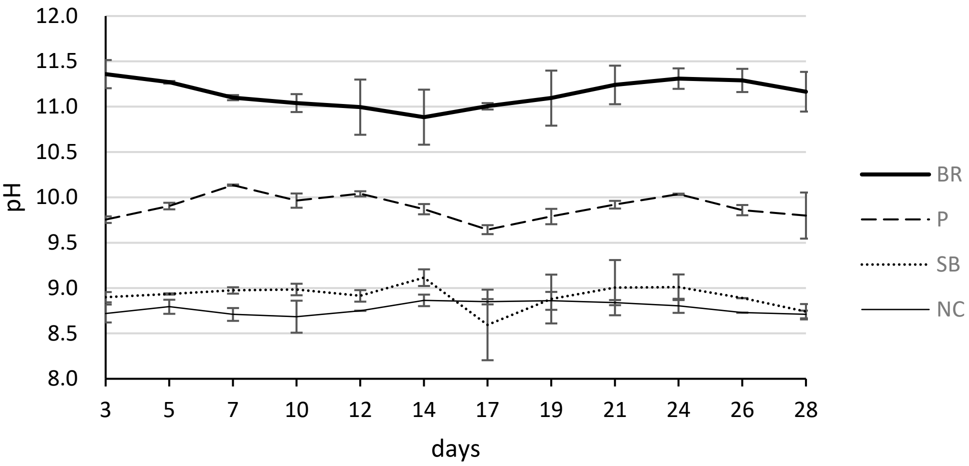

Figure S2. Changes in pH over time across different treatment groups. The pH values were measured on days 3, 5, 7, 10, 12, 14, 17, 19, 21, 24, 26, and 28. Groups include BR (solid line), P (long dashed line), SB (dotted line), and NC (thin solid line). Data are presented as mean ± standard deviation (SD).

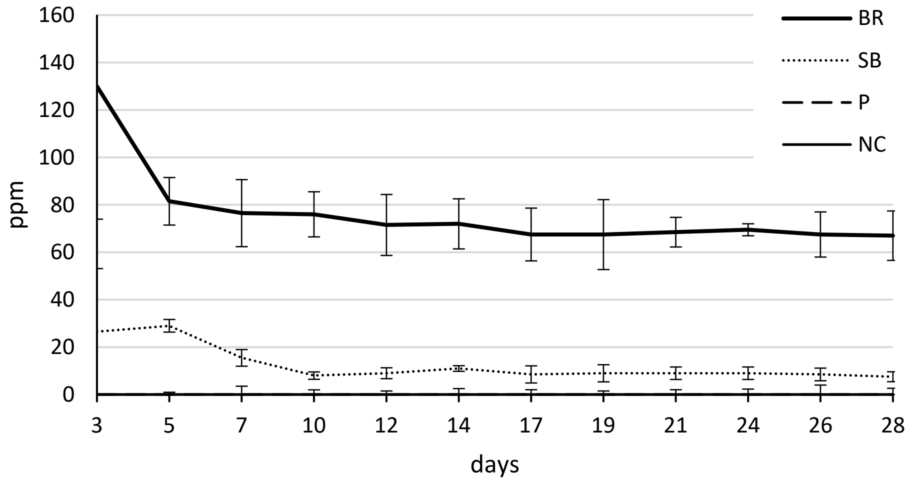

Figure S3. Changes in calcium ion over time across different treatment groups. The pH values were measured on days 3, 5, 7, 10, 12, 14, 17, 19, 21, 24, 26, and 28. Groups include BR (solid line), P (long dashed line), SB (dotted line), and NC (thin solid line). Data are presented as mean ± standard deviation (SD).

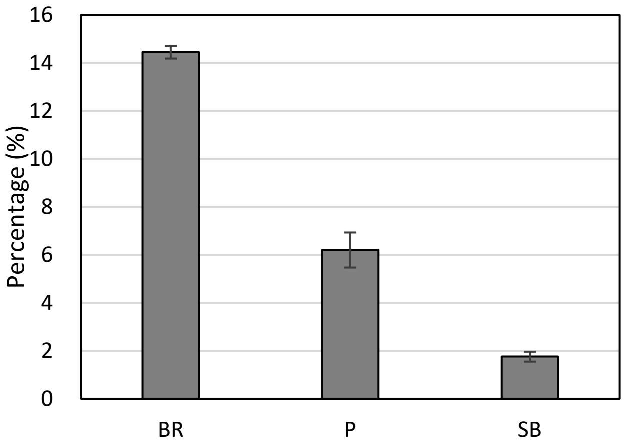

Figure S4. Mass change of the tested materials. The bars represent the mean percentage change in mass for BR, P, and SB after 28 days of immersion in deionized water. Error bars indicate the standard deviation
